# Src Drives Growth of Antiestrogen Resistant Breast Cancer Cell Lines and Is a Marker for Reduced Benefit of Tamoxifen Treatment

**DOI:** 10.1371/journal.pone.0118346

**Published:** 2015-02-23

**Authors:** Sarah L. Larsen, Anne-Vibeke Laenkholm, Anne Katrine Duun-Henriksen, Martin Bak, Anne E. Lykkesfeldt, Tove Kirkegaard

**Affiliations:** 1 Breast Cancer Group, Cell Death and Metabolism, Danish Cancer Society Research Center, Copenhagen, Denmark; 2 Department of Pathology, Slagelse Hospital, Slagelse, Denmark; 3 Statistics, Bioinformatics and Registry, Danish Cancer Society Research Center, Copenhagen, Denmark; 4 Department of Pathology, Odense University Hospital, Odense, Denmark; II Università di Napoli, ITALY

## Abstract

The underlying mechanisms leading to antiestrogen resistance in estrogen-receptor α (ER)-positive breast cancer is still poorly understood. The aim of this study was therefore to identify biomarkers and novel treatments for antiestrogen resistant breast cancer. We performed a kinase inhibitor screen on antiestrogen responsive T47D breast cancer cells and T47D-derived tamoxifen and fulvestrant resistant cell lines. We found that dasatinib, a broad-spectrum kinase inhibitor, inhibited growth of the antiestrogen resistant cells compared to parental T47D cells. Furthermore western blot analysis showed increased expression and phosphorylation of Src in the resistant cells and that dasatinib inhibited phosphorylation of Src and also signaling via Akt and Erk in all cell lines. Immunoprecipitation revealed Src: ER complexes only in the parental T47D cells. In fulvestrant resistant cells, Src formed complexes with the Human Epidermal growth factor Receptor (HER)1 and HER2. Neither HER receptors nor ER were co-precipitated with Src in the tamoxifen resistant cell lines. Compared to treatment with dasatinib alone, combined treatment with dasatinib and fulvestrant had a stronger inhibitory effect on tamoxifen resistant cell growth, whereas dasatinib in combination with tamoxifen had no additive inhibitory effect on fulvestrant resistant growth. When performing immunohistochemical staining on 268 primary tumors from breast cancer patients who had received tamoxifen as first line endocrine treatment, we found that membrane expression of Src in the tumor cells was significant associated with reduced disease-free and overall survival. In conclusion, Src was identified as target for treatment of antiestrogen resistant T47D breast cancer cells. For tamoxifen resistant T47D cells, combined treatment with dasatinib and fulvestrant was superior to treatment with dasatinib alone. Src located at the membrane has potential as a new biomarker for reduced benefit of tamoxifen.

## Introduction

Tamoxifen is recommended as first-line endocrine therapy for premenopausal women with estrogen receptor α (ER)-positive breast cancer [1]. Although many patients benefit from tamoxifen, *de novo* or acquired resistance occurs in ∼30% of patients after 15 years of follow up [1]. Upon progression, many patients respond to the pure antiestrogen fulvestrant (ICI 182,780 or faslodex) [2]. While tamoxifen is a selective ER modulator with partial ER agonistic activity, fulvestrant is a selective ER down modulator with pure ER antagonistic activity [3]. However, as for tamoxifen, resistance to fulvestrant is inevitable for patients with advanced disease. The underlying mechanisms for antiestrogen resistant breast cancer are still poorly understood. However, strong evidence implicates the involvement of cross-talk between ER, growth factor receptors and downstream signaling pathways [4]. To explore the resistance mechanisms, we have, by long-term treatment of the ER-positive breast cancer cell line T47D with fulvestrant or tamoxifen, established antiestrogen resistant cell lines [5,6]. We found that the tamoxifen resistant T47D cells remained ER-positive and could be growth inhibited by fulvestrant, indicating that at least part of the growth is mediated by ER [6]. In contrast, the fulvestrant resistant T47D cells were ER-negative but over expressed the Human Epidermal growth factor Receptor (HER)2. However, although HER2-over expressing, the HER receptors did not play a significant role for fulvestrant resistant growth. Instead, increased expression and phosphorylation of the Src family of intracellular non-receptor protein tyrosine kinases was seen in the fulvestrant resistant T47D cell lines and Src was identified as a driver for fulvestrant resistant cell growth [5].

Src is important for many intracellular processes including proliferation, differentiation, survival, migration and angiogenesis. Src interacts with a variety of different signaling molecules including growth factor receptors (e.g. HER receptors, platelet-derived growth factor receptor (PDGFR), fibroblast growth factor receptor (FGFR)), ephrins, cell-cell adhesion molecules, integrins and steroid receptors like ER [7,8]. Thus, Src plays a role in intracellular signaling and cross-talk between growth promoting pathways such as signaling via ER and growth factor receptors. The cellular localization of Src is essential for the function of the protein. Inactive Src is located in the cytoplasm and at perinuclear sites, whereas activated Src is localized at the plasma membrane [9]. The precise mechanism for the action of Src in cancer is still not fully elucidated. However, *in vitro* studies have shown that MCF-7 cells expressing high levels of activated Src are more invasive [10], and that tamoxifen resistance in MCF-7 cells is accompanied by increased Src activity [11]. Combined targeting of Src and ER completely abrogates the invasive behavior of tamoxifen resistant MCF-7 and T47D breast cancer cell lines [12] and reduces cell growth and survival of long-term estrogen deprived (LTED) cells [13]. Compared with normal breast tissue, Src expression and activity is increased in breast cancers [14–16], and increased Src activity is associated with higher risk of recurrence in ER-positive disease [17,18]. The majority of breast cancers with over expressed or activated Src also over express one of the HER receptors [16,19], and in HER2-positive breast cancer, activated Src correlates with HER2 positivity and poor prognosis [20]. Thus, Src is identified as a converging point of multiple resistance mechanisms and targeting Src might therefore be a promising therapeutic approach in solid tumors. The broad-spectrum tyrosine kinase inhibitor dasatinib (BMS-354825; Bristol-Myers Squibb) has so far been the most clinically studied Src inhibitor [21]. Dasatinib was initially identified as a dual Src and Bcr/Abl inhibitor and is approved for the treatment of imatinib-resistant chronic myeloid leukemia [22,23]. Recently, however, preclinical experiments have provided the bases for investigating dasatinib as a targeted therapy in a variety of solid tumors including breast cancers [24].

One of the key issues in the treatment of ER-positive breast cancers is the ability to predict whether first-line adjuvant endocrine therapy alone is sufficient to reduce the risk of relapse, or if the patient should be offered additional or alternative treatment e.g. treatment combining endocrine and non-endocrine agents. To explore this, studies into the molecular mechanisms behind acquired antiestrogen resistance are essential. In this study we have utilized a kinase inhibitor library to identify kinases driving growth of our antiestrogen resistant T47D breast cancer cell lines. Dasatinib was identified as a kinase inhibitor targeting growth of both fulvestrant and tamoxifen resistant cells. The role of Src in signaling and growth of the resistant cells was therefore explored using dasatinib alone and in combination with the antiestrogens. Moreover, the clinical relevance of Src in tumors from tamoxifen-treated breast cancer patients was investigated.

## Materials and Methods

### Cell lines, culture conditions and reagents

The T47D cell line was originally obtained from the Human Cell Culture Bank (Mason Research Institute, Rockville, MD, USA) and maintained as previously described [5]. The fulvestrant resistant cell lines, T47D/182^R^-1 (182^R^-1) and T47D/182^R^-2 (182^R^-2) were established by long term treatment with 100 nM fulvestrant as described in [5]. To enable ER-mediated growth inhibition by tamoxifen, the T47D cell line was adopted to grow in standard growth medium supplemented with only 2% FBS. Establishment of the tamoxifen resistant cell lines T47D/TR-1 (TR-1) and T47D/TR-2 (TR-2) is described in [6]. The fulvestrant and tamoxifen resistant cell lines were maintained in the same growth medium as the parental T47D cell lines plus 1 μM tamoxifen (Sigma-Aldrich, Copenhagen, Denmark) or 100 nM fulvestrant (Tocris, Avonmouth, Bristol, UK), respectively. For experiments, 2.5 x 10^5^ U penicillin and 31.25 μg/l streptomycin (Gibco, Invitrogen, CA, USA) were added to the growth medium. Parental T47D cell lines grown in 2% and 5% FBS will be referred to as T47D/S2 and T47D/S5, respectively.

### Kinase screen

The kinase inhibitor library comprising 195 different kinase inhibitors was purchased from Selleck Chemicals (Houston, TX, USA). Cells were seeded in triplicate in 96-well plates in standard growth medium and allowed to adhere for two days before five days treatment with 1 μM concentration of the kinase inhibitors. Vehicle-treated (0.1% DMSO) controls were included in each plate (6–10 wells/plate). Cell viability was assayed using CellTiter-Glo Luminescent Cell Viability Assay (Promega, Madison, WI, USA) and luminescence was measured using Varioscan Flash platereader (Thermo Scientific, Waltham, MA, USA).

### Cell growth assays

Cells (3000–4800 cells/well) were seeded in 96-well plates in their respective growth medium and allowed to adhere for two days prior to five days treatment. Cell number was determined using a crystal violet colorimetric assay as previously described [25]. For experiments where the effect of dasatinib, tamoxifen and fulvestrant alone and in combinations was investigated on cell growth, antiestrogens were withdrawn from the resistant cell culture medium one week prior to onset of experiment. To investigate if tamoxifen resistant cell lines could be propagated during long-term treatment in the presence of dasatinib and/or antiestrogens, 4x10^5^ cells (T47D/S2, TR-1 and TR-2) withdrawn from tamoxifen were seeded in T25-flasks. Experimental medium with dasatinib (1 μM), tamoxifen (1 μM) and fulvestrant (100 nM) alone or in combination were added two days after plating. After 7 days, growth rate was determined as the ratio between cell number after seven days treatment and number of seeded cells. From each culture, 4x10^5^ cells were reseeded in T25-flasks and grown for further two weeks with determination of cell number each week. When fewer than 4x10^5^ cells were counted after one week, the total number of cells were seeded and used for calculations.

### Western blot analysis

Western blot analysis was performed as previously described [5]. The following antibodies were purchased from Cell Signaling Technology (Beverly, MA, USA); phosphorylated Akt (Ser^473^, 1:500, #9271, Rabbit polyclonal), phosphorylated Erk (Thr^202^/Tyr^204^, 1:1000, #4377, Rabbit monoclonal), phosphorylated Src (Tyr^416^, 1:100, #2101, Rabbit polyclonal), Akt (1:2000, #9272, Rabbit polyclonal), Erk (1:2000, #9102, Rabbit polyclonal) and Src (1:1000, #2109, Rabbit polyclonal). Hsp70 (1:500.000, #MS-482, Mouse monoclonal) were purchased from Thermo Scientific and β-actin (1:500.000, #A5441, Mouse monoclonal) from Sigma Aldrich. The experiments were performed at least twice and representative blots are shown.

### Immunoprecipitation

Cell lysates were generated with cell lysis buffer from Cell Signaling Technology (#9803) and immunoprecipitation performed as previously described [5]. Antibodies against the following proteins were used; HER1 (1:1000, #M7298, mouse monoclonal) purchased from Dako, HER2 (1:2000, #MS-730, Mouse monoclonal) and HER3 (1:2000, #MS-201, Mouse monoclonal) purchased from Thermo Scientific, and Src (1:1000, #2109, Rabbit polyclonal) and ER (1:1000, #2512, Mouse monoclonal) purchased from Cell Signaling Technology. As input control, 20 μg of total protein was analyzed. The experiments were performed twice.

### Patients

The cohort included 268 high-risk postmenopausal breast cancer patients diagnosed between 1989 and 2001 and treated with tamoxifen as first-line adjuvant endocrine treatment according to guidelines from the Danish Breast Cancer Cooperative Group (DBCG) [26]. Archival formalin-fixed and paraffin-embedded breast cancer tissue was previously collected from the archive at the Pathology Department at Odense University Hospital. Tissue micro arrays (TMAs) were generated using two 2 mm cores from each patient. The cores were representative invasive tumor tissue identified by marking the corresponding tumor area on haematoxylin and eosin stained sections from each paraffin block. Standard clinico-pathological parameters have previously been shown [27]. The biomarker study was approved by the local Ethical Committee for Region South Denmark (S-VF-20040064), the Ethical Committee waived the requirement for informed consent from the participants. Patient tissue and data were anonymized throughout the study.

### Immunohistochemistry (IHC)

IHC was conducted on tissue microarrays (TMAs) using a standard immunoperoxidase procedure [27–29]. In brief, antigen retrieval was performed by microwaving the TMA slides, comprising two 2 mm cores from each patient, in citrate-buffer, pH 6.0 (Dako). Endogenous peroxidase activity was quenched by 3% hydrogen peroxide (H_2_O_2_) and non-specific binding blocked by Serum-free protein block (Dako). Primary antibodies for total (1:300, #2109, Cell Signaling Technology) and phosphorylated (1:50, #2101, Cell Signaling Technology) Src were applied at 4°C ON. Envision (Dako) was used for signal amplification and positive staining was visualized by 3.3-diaminobenzidine tetrahydrochloride (Dako). Nuclei were counterstained with haematoxylin and the slides mounted in pertex (Histolab, Göteborg, Sweden).

### Evaluation of IHC

Evaluation of the markers was performed blindly without any prior knowledge of patient history including clinical data or other histological findings. Total and phosphorylated cytoplasmic and membrane Src protein expression of each TMA core was manually assessed according to the weighted histoscore (cytoplasmic) [30] and HercepTest (membrane) guidelines. All tumors were scored by TK whereas A-VL scored 25% of the tumors.

### Statistical analysis

For the kinase inhibitor screen one-tailed Student’s *t*-test was performed on triplicate values comparing effect in parental and resistant cell lines. For cell growth assays, two-tailed unequal variance t-test followed by Bonferroni’s correction was used. In the clinical study, uni- and multivariate analyses were performed. The multivariate analysis included tumor grade, size, nodal status and age as standard covariates. Kaplan-Meier life tables with log-rank testing were generated to assess the effect of membrane-localized Src on disease-free and overall survival. To investigate the association between tumor expression of Src and HER2-positivity or expression of activated HER receptors, χ^2^-test was used. The clinical analyses were performed in R version 3.0.1 with the R package “rms”. P<0.05 was considered statistically significant.

## Results

### Kinase inhibitor screen identifies dasatinib to preferential inhibit growth of both tamoxifen and fulvestrant resistant cells

**Fig 1:**
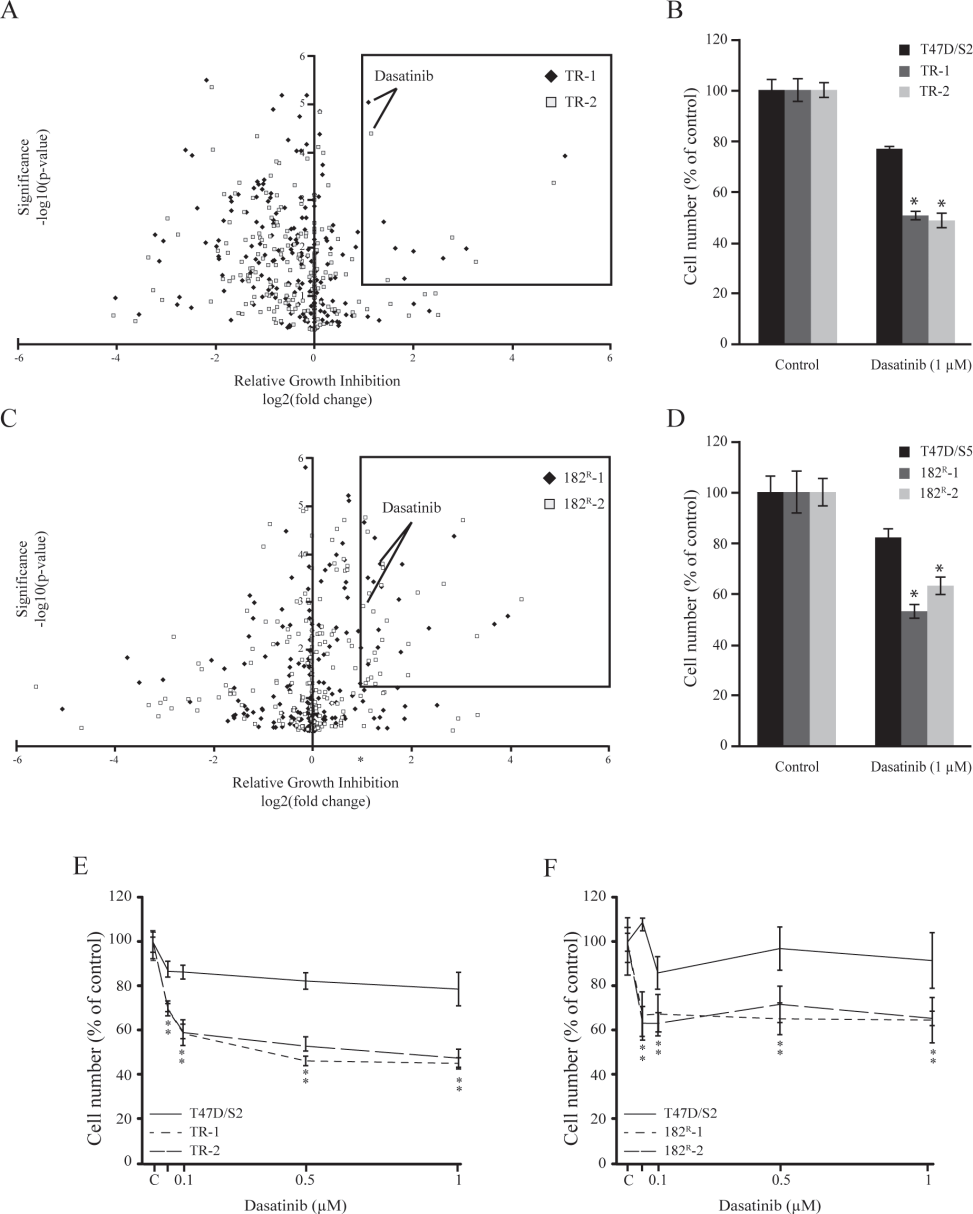
Identification of dasatinib as a kinase driving growth of tamoxifen and fulvestrant resistant breast cancer cell lines. (A, C) Parental (T47D/S2 and T47D/S5), tamoxifen (TR-1 and TR-2) and fulvestrant (182^R^-1 and 182^R^-2) resistant cell lines were treated for 5 days with a kinase inhibitor library comprising 195 different kinase inhibitors (1 μM). Cell number was assessed by a CellTiter-Glo Luminescent Cell Viability Assay. Volcano plots are displaying statistical significance (P<0.05) versus fold change in relative growth inhibition between parental and resistant cells. The boxes indicate kinases with more than two-fold preferential growth inhibition of the resistant cell lines compared to their parental T47D cells and P<0.05. (B, D) Mean cell numbers of parental and resistant cells treated with 1 μM dasatinib shown as percentage of untreated control. The results are from the kinase inhibitor screens. (E, F) Parental and resistant cell lines were treated for 5 days with the indicated concentrations of dasatinib. Cell number was measured by a colorimetric assay and expressed as percentage of untreated control (designated C). A representative experiment with mean ± SD is shown. * P<0.05 for dasatinib treated samples vs control.

To identify kinases causally involved in antiestrogen resistant breast cancer, parental (T47D/S2 and T47D/S5), tamoxifen (TR-1 and TR-2) and fulvestrant resistant (182^R^-1 and 182^R^-2) breast cancer cell lines were subjected to a kinase inhibitor screening library containing 195 different kinase inhibitors each targeting one or more kinases. Upon five days treatment, several kinase inhibitors were identified to preferentially inhibit growth of the antiestrogen resistant T47D cell lines compared to the parental cells. The results from the screens are shown in volcano plots, displaying statistical significance (P<0.05) versus fold change in relative growth inhibition between parental and resistant cells (Fig. 1A, C). We wanted to identify inhibitors which preferentially targeted growth of both tamoxifen and fulvestrant resistant cell lines, with a statistically significant growth inhibition which was at least two-fold higher than the inhibition of parental cells. The only inhibitor which fulfilled these criteria was the broad-spectrum tyrosine kinase inhibitor, dasatinib (Fig. 1A, C). In the screen, 1 μM dasatinib exhibited 40–50% growth inhibition of both tamoxifen and fulvestrant resistant cell lines compared to 18% and 23% growth inhibition of the parental T47D/S5 and T47D/S2 cells, respectively (Fig. 1B, D).

**Fig 2:**
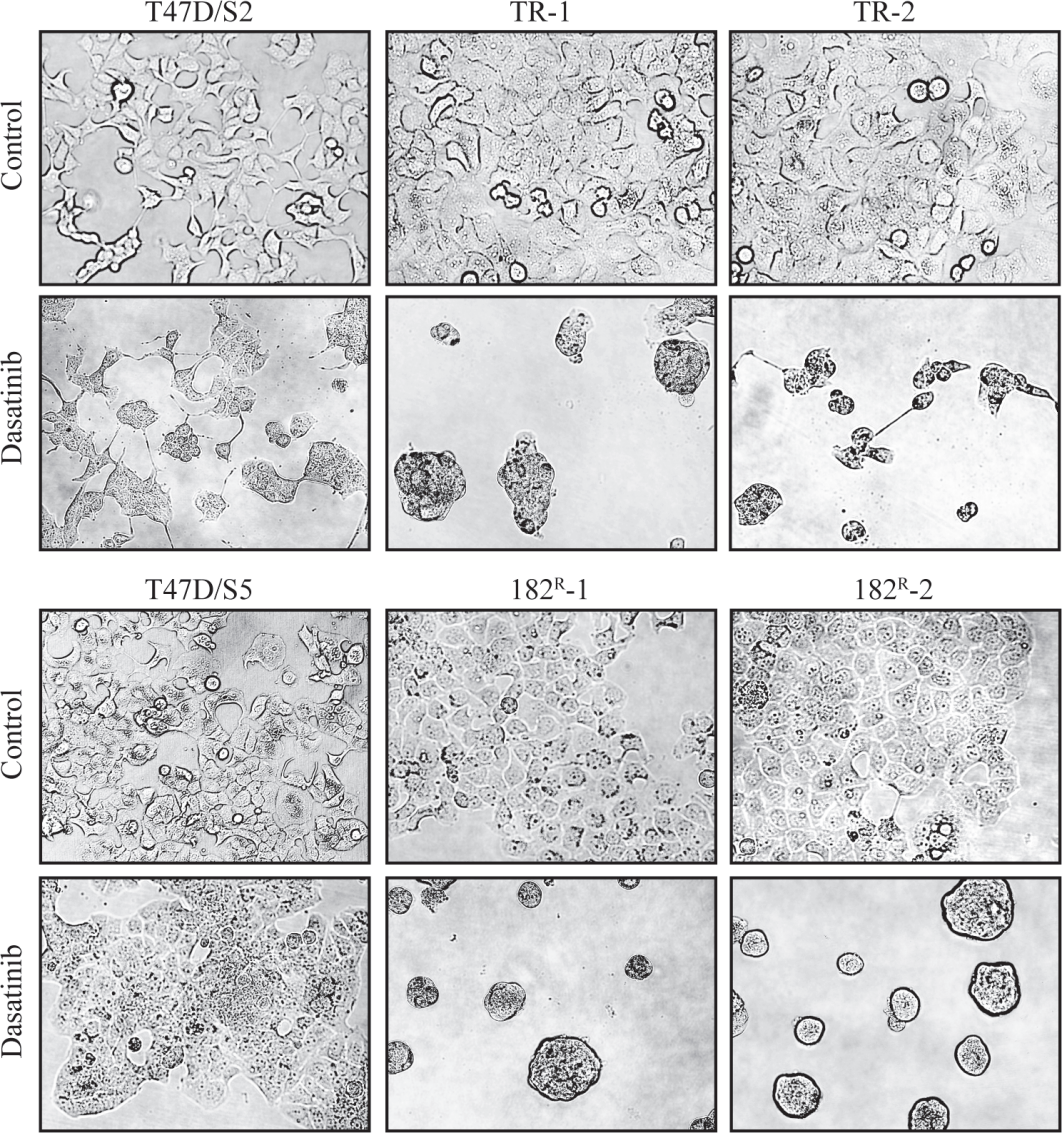
Effect of dasatinib on the morphology of parental and resistant cells. Representative pictures of parental (T47D/S2 and T47D/S5), tamoxifen (TR-1 and TR-1) and fulvestrant (182^R^-1 and 182^R^-2) resistant cell lines treated for five days with dasatinib (1 μM) or DMSO (control).

To validate the growth inhibitory effects seen in the kinase inhibitor experiments, dose-response growth experiments were performed. Parental, tamoxifen and fulvestrant resistant cell lines were treated with increasing concentration of dasatinib (0.05–1 μM). Dasatinib exerted a dose-dependent growth inhibition, and 0.1 μM exerted close to maximal effect of approximately 60% and 40% for tamoxifen and fulvestrant resistant cell lines, respectively, compared to the untreated controls (Fig. 1E, F). In contrast, the maximal growth inhibitory effect of dasatinib on the parental T47D cell lines was only 20%. Thus, the dose-response growth experiments confirmed that the resistant cell lines were more sensitive to dasatinib compared to the parental cells. Visual inspection of the morphology of the cells upon treatment with dasatinib (1 μM) revealed substantial differences between parental and resistant cells. For both tamoxifen and fulvestrant resistant cell lines, the ability to adhere was severely affected. The cells detached and formed spheres containing multiple cells. In contrast, the morphology of the parental cell lines was much less affected by treatment with dasatinib (Fig. 2).

### Dasatinib prevents phosphorylation of Src and downstream signaling molecules in parental and resistant T47D cell lines

**Fig 3:**
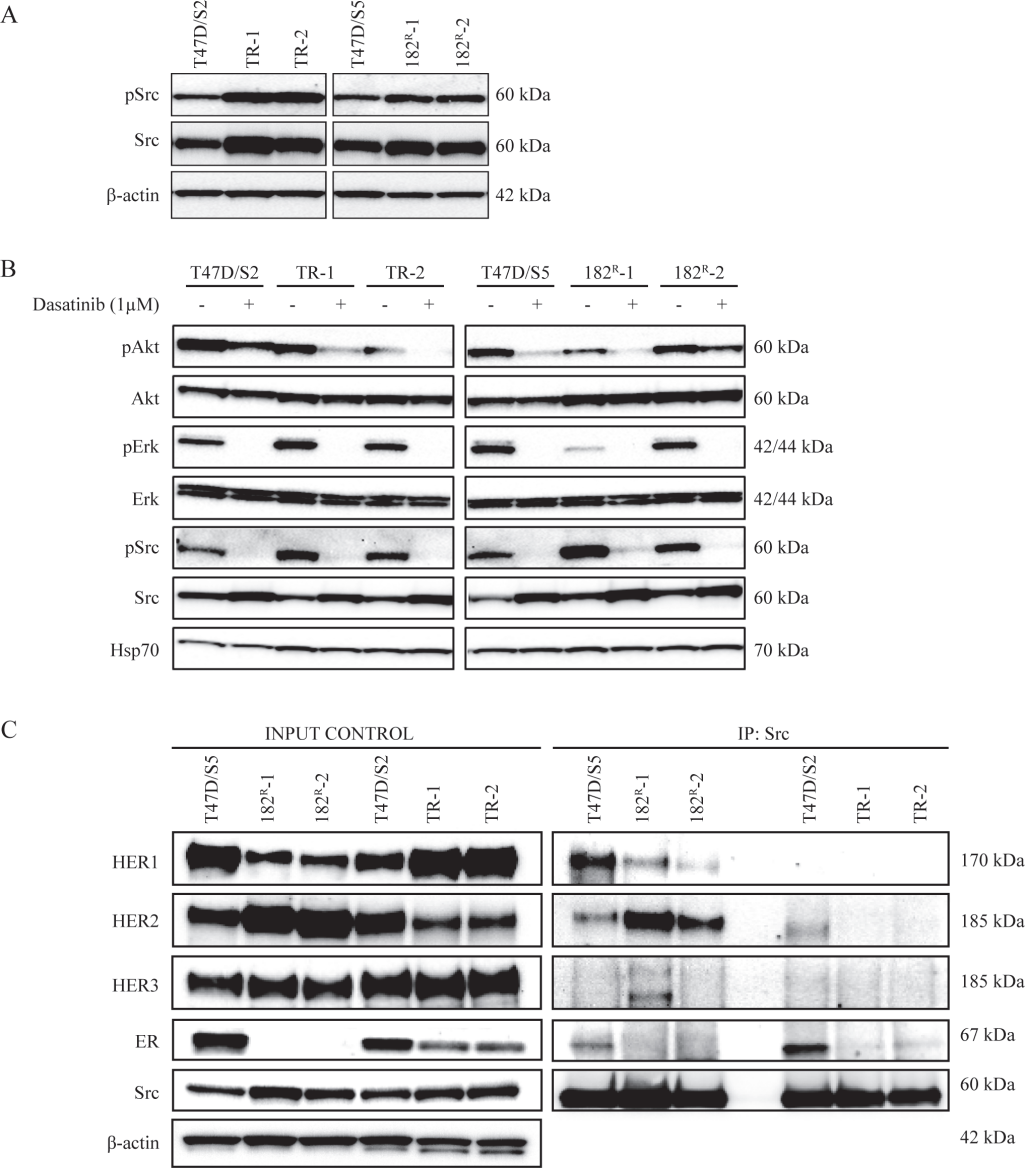
Effect of dasatinib on expression of Src and downstream signaling in parental and resistant cells and identification of Src dimers. (A) Western blot showing expression of total and phosphorylated Src (Tyr^416^) in lysates from parental (T47D/S2 and T47D/S5), tamoxifen (TR-1 and TR-2) and fulvestrant (182^R^-1 and 182^R^-2) resistant cell lines grown under their standard conditions. β-actin was used as loading control. (B) Western blot showing expression of total and phosphorylated Akt (Ser^473^), Erk (Thr^202^/Tyr^204^) and Src (Tyr^416^) in lysates from parental (T47D/S2 and T47D/S5), tamoxifen (TR-1 and TR-2) and fulvestrant (182^R^-1 and 182^R^-2) resistant cell lines grown with or without dasatinib (1 μM) for 1 hour. Hsp70 was used as loading control. (C) Src dimers in parental (T47D/S2 and T47D/S5), tamoxifen (TR-1 and TR-2) and fulvestrant (182R-1 and 182R-2) resistant cell lines grown under their standard conditions. Src protein was immunoprecipitated from 1 mg total protein lysates. Western analysis for HER receptors, Src and ER was performed on both total protein lysates and immunoprecipitated samples. A representative experiment out of two is shown. β-actin was used as loading control.

Compared with normal breast tissue, Src activity is increased in human breast tumors [14,15]. Therefore we investigated Src expression and activation in the resistant cell lines. Expression and phosphorylation of Src was increased in all antiestrogen resistant T47D breast cancer cell lines compared to the level in the parental cell lines (Fig. 3A). Activation of growth factor receptors and downstream signaling pathways such as the PI3K-Akt and Ras/Raf/MAPK pathways is known to be involved in the development of acquired resistance to antiestrogens [31–36]. Thus, to investigate the effect of dasatinib on signaling in the parental and resistant T47D breast cancer cell lines, expression and phosphorylation of Src, Akt and Erk were measured by western analysis. We found that 1 μM dasatinib completely blocked phosphorylation of Erk at Thr42/Tyr44 and Src at Tyr416 in parental and resistant cell lines, whereas phosphorylation of Akt at Ser473 was clearly reduced. The expression of total Akt and Erk was unchanged and the expression of total Src increased in all cell lines upon treatment with dasatinib (Fig. 3B). Taken together, dasatinib inhibits signaling via Src, Akt and Erk in parental and antiestrogen resistant T47D breast cancer cell lines.

### Src forms complexes with HER receptors in the parental and fulvestrant resistant cell lines

In our previous study we found that Src formed complex with HER2 in the fulvestrant resistant T47D cell lines [5]. Other *in vitro* studies have shown that the association between Src and HER receptors is critical for HER signaling [37]. We therefore used immunoprecipitation experiments to explore complex formation between Src and HER receptors. In agreement with our previous study [5], the basal expression of HER1 was decreased, HER2 expression increased and HER3 expression unchanged in fulvestrant resistant T47D breast cancer cell lines compared to the parental T47D/S5 cells (Fig. 3C). The parental T47D/S5 cells expressed ER, whereas the fulvestrant resistant cells were ER-negative [5]. Compared to parental T47D/S2 cells, the tamoxifen resistant cell lines had increased expression of HER1, decreased expression of HER2 and unchanged HER3 expression. ER expression was decreased in the tamoxifen resistant cells compared to the expression in parental T47D/S2. HER1 and HER2 were co-precipitated with Src in T47D/S5 and the fulvestrant resistant cell lines, and the levels reflected the expression in total lysates. In T47D/S2, a small amount of HER2 was precipitated with Src whereas none of the HER receptors formed complex with Src in the tamoxifen resistant cell lines (Fig. 3C). Src was only co-precipitated with ER in the parental cells (Fig. 3C). Thus, in the fulvestrant resistant cells, Src was associated with HER1 and HER2, whereas immunoprecipitation of Src from the tamoxifen resistant cell lines did not reveal complex formation between Src and any of the HER receptors or ER.

### Dasatinib in combination with fulvestrant exerts superior growth inhibition compared to single agent treatment in tamoxifen resistant cells

**Fig 4:**
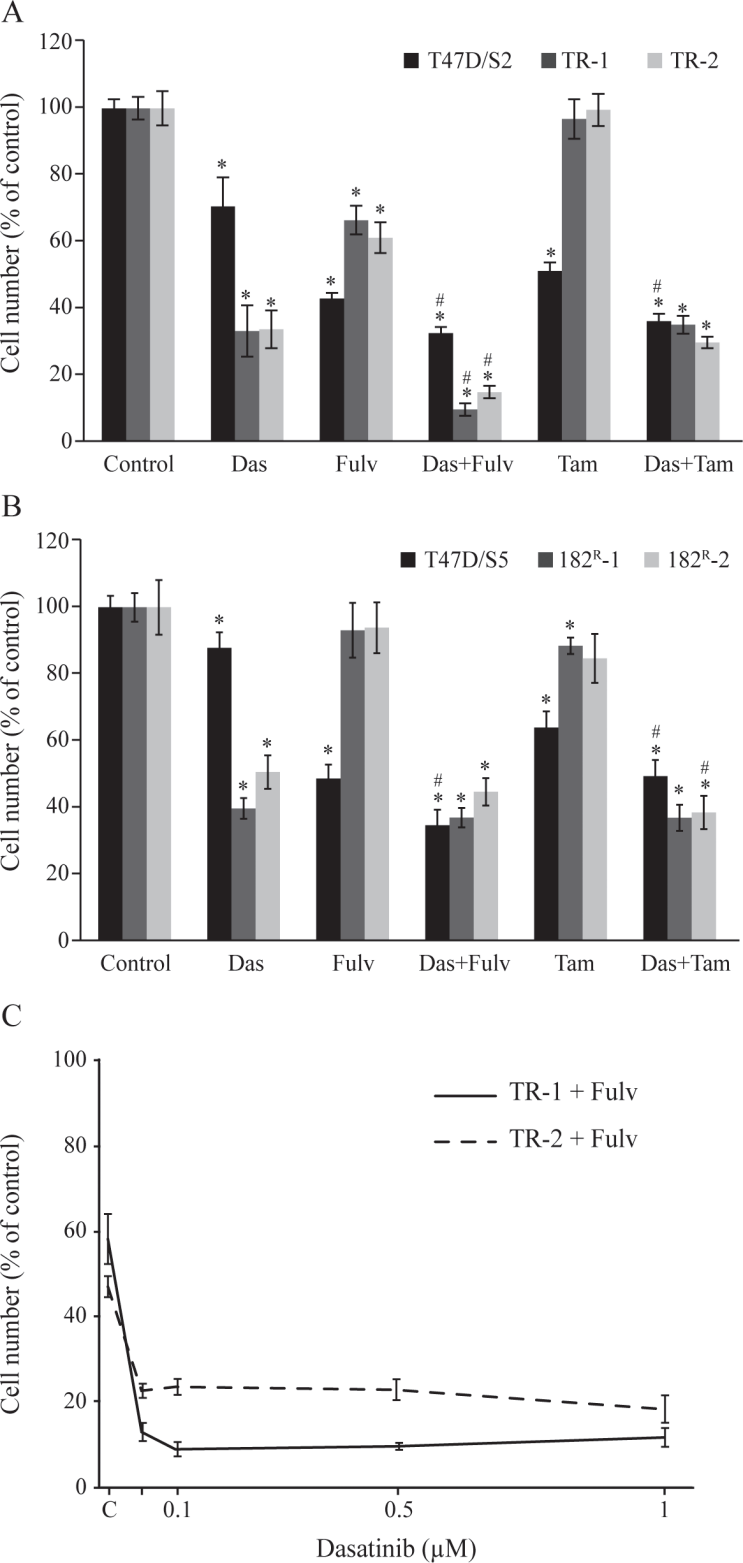
Effect of dasatinib, tamoxifen and fulvestrant on growth of parental and resistant T47D cells. (A, B) Parental and resistant cell lines were grown for 5 days with vehicle (0.1% DMSO), 1 μM dasatinib (Das), 100 nM fulvestrant (Fulv) or 1 μM tamoxifen (Tam) alone or in the indicated combinations. (C) Tamoxifen resistant cell lines were treated for five days with 100 nM fulvestrant and dasatinib at the indicated concentrations. Cell number was measured by a crystal violet colorimetric assay and expressed as percentage of untreated control. A representative experiment out of three with mean ± SD is shown. * P<0.05 for treated vs untreated cells; # P<0.05 for cells treated with dasatinib vs cells treated with a combination of dasatinib and tamoxifen or fulvestrant.

Combined treatment targeting signaling via ER and Src has been suggested as a future treatment option of patients with endocrine resistant breast cancer [13,38]. In order to investigate whether combined treatment with dasatinib and antiestrogens could enhance growth inhibition in our resistant cells, cells were treated for five days with fulvestrant (100 nM), tamoxifen (1 μM) and dasatinib (1 μM) alone or in combination. Dasatinib alone inhibited growth of parental T47D/S2 and T47D/S5 cells to 70% and 90%, respectively, of vehicle treated cells (Fig. 4A, B). Treatment of parental cells with tamoxifen or fulvestrant alone inhibited growth to 40–60% of vehicle treated controls (Fig. 4A, B). Combined treatment with dasatinib and either tamoxifen or fulvestrant had an additional growth inhibitory effect of the parental cells to 35–40% of the growth of vehicle treated control (Fig. 4A, B). For tamoxifen resistant T47D cell lines, dasatinib alone inhibited growth to 35% of untreated controls, whereas dasatinib combined with fulvestrant resulted in almost complete growth inhibition (Fig. 4A). Notably, concentrations as low as 0.1 μM dasatinib in combination with fulvestrant were able to totally arrest growth of tamoxifen resistant cell lines (Fig. 4C). As expected, treatment of the tamoxifen resistant cell lines with dasatinib in combination with tamoxifen had no additional growth inhibitory effect compared to treatment with dasatinib alone (Fig. 4A). For fulvestrant resistant cells, treatment with dasatinib alone inhibited growth to 40–50% of vehicle treated controls and combined treatment of dasatinib with either tamoxifen or fulvestrant exerted no additional growth inhibitory effects (Fig. 4B). Collectively, these data show that growth of tamoxifen resistant cell lines can be almost fully prevented by combined treatment with fulvestrant and dasatinib whereas addition of tamoxifen did not further increase the growth inhibitory effect of dasatinib on fulvestrant resistant cell lines.

### Combined treatment with antiestrogens and dasatinib prevents long-term propagation of tamoxifen resistant cell lines

**Fig 5:**
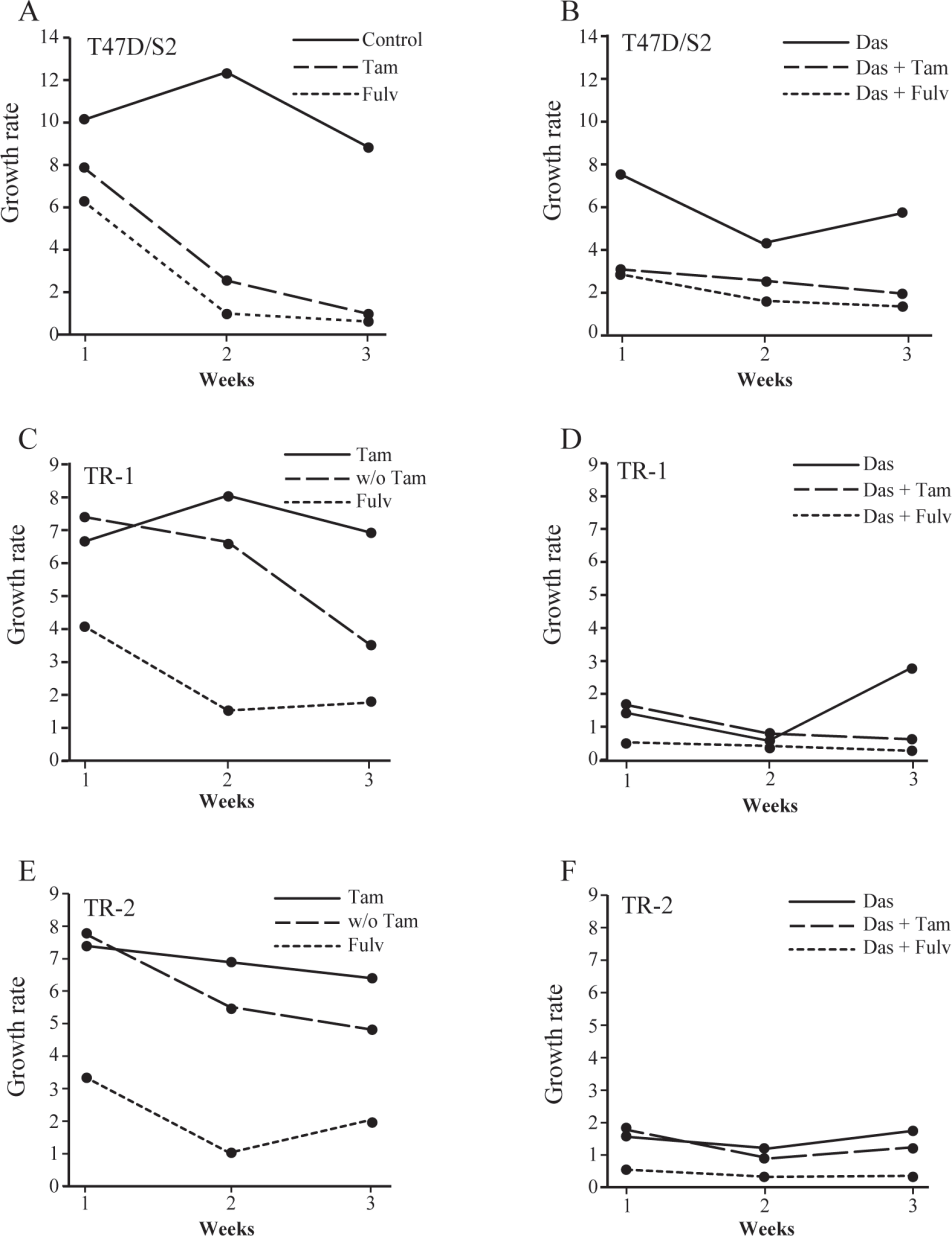
Effect of dasatinib, tamoxifen and fulvestrant alone or in the indicated combinations on long-term propagation of parental and tamoxifen resistant cell lines. T47D/S2 (A, B), TR-1 (C, D) and TR-2 (E, F) cells were seeded with 4x10^5^ cells in T25-flasks and treated for one week with 1 μM dasatinib (Das), 1 μM tamoxifen (Tam) or 100 nM fulvestrant (Fulv) alone or in combination as indicated in the figure. Control cells were propagated in medium containing DMSO and/or ethanol (vehicle) corresponding to the same amount as treated cultures. Growth rate was determined as the ratio between cell number after one week treatment and number of seeded cells. After cell number determination, 4x10^5^ cells were reseeded in T25-flasks and allowed to grow for additional two weeks with weekly split and determination of cell number. In cases with fewer than 4x10^5^ cells, the total number of cells were added and used for the calculations. w/o; without.

In order to explore the effect of antiestrogens and dasatinib on cell propagation, tamoxifen resistant cells were treated with fulvestrant (100 nM), tamoxifen (1 μM) and dasatinib (1 μM), alone or in combination, and the ability of the cells to propagate continuously was investigated. The cells were grown for a total of three weeks in the presence of antiestrogens and/or dasatinib. Every week cell number was determined and growth rate calculated as the ratio between cell number after one week of growth and number of seeded cells. Growth rate of parental T47D/S2 was around 10, whereas TR-1 and TR-2 had a growth rate around 7 in their standard medium with 1 μM tamoxifen (Fig. 5A, C, E). Treatment with tamoxifen or fulvestrant alone or in combination with dasatinib for 3 weeks, decreased the growth rate of parental T47D/S2 cells 5–10 times compared to the growth rate of control cells (Fig. 5A, B). In contrast, T47D/S2 grown in the presence of dasatinib alone only had a slightly decreased growth rate compared to control cells (Fig. 5A, B). Growth rate of the tamoxifen resistant cells was clearly decreased in the absence of tamoxifen (Fig. 5C, E). Treatment with fulvestrant and dasatinib alone or dasatinib in combination with tamoxifen reduced, but did not block, growth of TR-1 and TR-2. Noteworthy, after one week of combined treatment of tamoxifen resistant cells with fulvestrant and dasatinib, cell number was reduced to 25% of the seeded amount of cells and no increase in cell number was observed after 2 and 3 weeks treatment (Fig. 5D, F). These data show that growth of tamoxifen resistant cell lines can be totally blocked by combined treatment with dasatinib and fulvestrant.

### Total and phosphorylated Src are localized in the cytoplasm and membrane of ER-positive breast tumors

**Fig 6:**
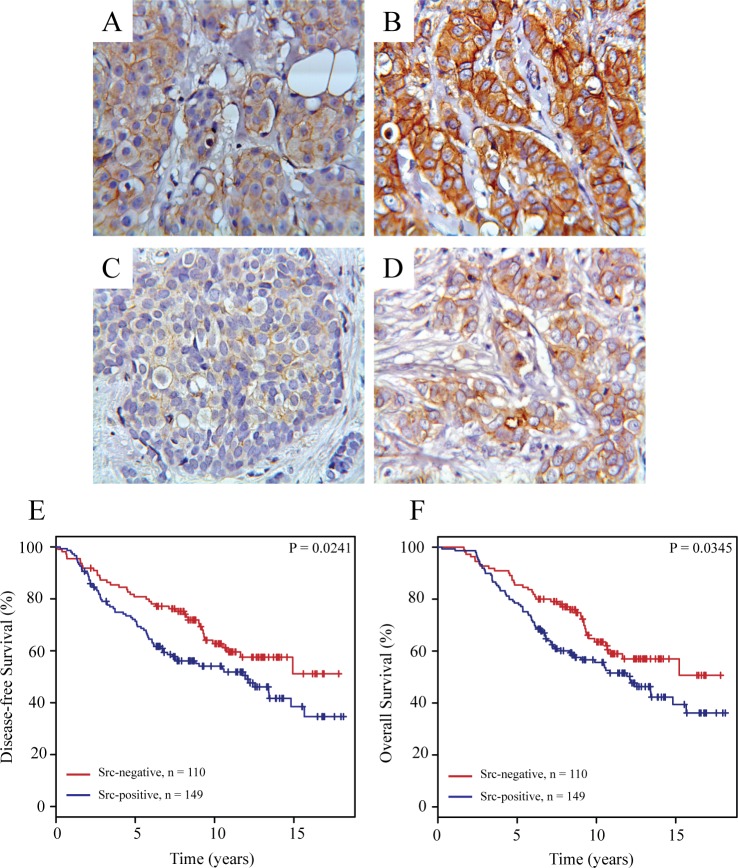
Immunohistochemical staining of Src expression and phosphorylation in primary breast tumors and Kaplan-Meier survival estimates relative to the expression of Src at the plasma membrane. Representative pictures of weak (A) and strong (B) immunohistochemical staining of total Src and of weak (C) and strong (D) immunohistochemical staining of phosphorylated Src (Tyr^416^) expressed in primary breast tumors. Kaplan-Meier survival plots demonstrating percentage disease-free (E) and overall (F) survival in patients with tumors with or without Src expressed at the plasma membrane.

To elucidate the clinical relevance of our findings that Src is important for growth and signaling in the antiestrogen resistant T47D breast cancer cell lines, we evaluated the subcellular localization of total and phosphorylated Src by immunohistochemistry in tumors from 268 high-risk breast cancer patients, who have received tamoxifen as first-line adjuvant endocrine treatment. Total and phosphorylated Src staining could be evaluated in 259 (97%) and 262 (98%) breast carcinomas, respectively. Cytoplasmic Src staining and staining of Src at the plasma membrane of the tumor cells were seen in 248 (95.7%) and 149 (57.5%) tumors, respectively, whereas phosphorylated Src was seen in the cytoplasm and at the plasma membrane of 91 (35%) and 36 (14%) tumors, respectively. Strong cytoplasmic Src staining was also observed in lymphocytes and in some ductal carcinoma *in situ* (DCIS). Fig. 6A-D shows examples of breast carcinomas with weak and strong staining of total and phosphorylated Src in the cytoplasm and at the plasma membrane.

### Src located at the plasma membrane is a potential biomarker for disease-free and overall survival of tamoxifen treated breast cancer patients

To explore the clinical importance of Src as a marker for disease-free and overall survival, cytoplasmic Src expression was divided into high (above median; ≥4% positive tumor cells) and low (below median; <4% positive tumor cells) staining, whereas expression of total and phosphorylated Src at the plasma membrane and expression of phosphorylated Src in the cytoplasm were divided into negative (no staining) and positive (any staining). Univariate analysis revealed a significant association between Src expressed at the plasma membrane and reduced disease-free and overall survival (P = 0.0042 and P = 0.0357, respectively). No significant association was observed for phosphorylated Src or cytoplasmic Src and disease-free or overall survival (data not shown). When analyzed by multivariate analysis including the standard covariates; tumor grade, tumor size, nodal status and age only high level of Src at the plasma membrane was a significant and independent biomarker for poor disease-free (P = 0.0042) and overall (P = 0.0268) survival. Kaplan-Meier life tables also showed a significant association between expression of Src at the plasma membrane and reduced disease-free (P = 0.0241) and overall (P = 0.0345) survival (Fig. 6E, F). For this cohort, 15-years disease-free and overall survival for patients with Src expressed at the plasma membrane were only 30% compared to 50% for patients with no Src expressed at the plasma membrane.

### Src expression at the plasma membrane is associated with HER2-positivity and activated HER receptors

Significant association between expression of Src and HER receptors has been observed in breast cancers [16,19]. We have previously described the expression of active HER receptors in tumors from the above-used cohort of tamoxifen-treated breast cancer patients [27]. We therefore investigated the correlation between Src expression at the plasma membrane and HER2-positivity or activated HER receptors, and found that expression of Src was significantly associated with both HER2 positivity and expression of activated HER1-3 in the breast tumors (Table 1).

**Table 1:** Expression of HER2, pHER1, pHER2 and pHER3 in tumors with or without Src expressed at the plasma membrane.

	Tumors without membrane Src (n = 110)	Tumors with membrane Src (n = 149)
HER2-positive	1.8% (n = 2)	12.8% (n = 19)
pHER1	13.6% (n = 15)	21.5% (n = 32)
pHER2	7.2% (n = 8)	19.5% (n = 29)
pHER3	9% (n = 10)	20% (n = 30)

## Discussion

Despite thorough investigation of the molecular mechanisms behind antiestrogen resistant breast cancer, it is still a major clinical challenge to treat patients with resistant disease. In this study we have subjected tamoxifen and fulvestrant resistant T47D breast cancer cell lines to a kinase inhibitor library and identified dasatinib as a potential new treatment option for antiestrogen resistant breast cancer. The broad-spectrum kinase inhibitor dasatinib was originally isolated as a dual Src and Bcr/Abl inhibitor [22,23]. Dasatinib targets Src family kinases in general as well as multiple receptor tyrosine kinases [13,24], and has been used in the treatment of leukemia. In our study, we observed close to maximum effect of dasatinib when using 0.1 μM. This concentration (48.8 μg/L) is within the range of the blood level concentration (1–143 μM) in patients treated with the recommended dose of 100 mg/day dasatinib and which exerts manageable side effects [39]. As Src activity has been linked to tamoxifen resistance, the possibility of using dasatinib to treat antiestrogen resistant breast cancer has been suggested [40]. In fact, clinical studies evaluating dasatinib in combination with letrozole (NCT00696072) or fulvestrant (NCT00903006) as a treatment option for ER-positive/HER2-negative metastatic breast cancer is ongoing (see www.clinicaltrial.gov).

In agreement with previous studies, we found increased Src expression and activation in our tamoxifen and fulvestrant resistant T47D breast cancer cell lines [11,40]. The finding that dasatinib is able to prevent signaling via Src and Erk, and to reduce signaling via Akt in both parental and resistant cell lines, may, at least in part, explain the observed inhibition of cell growth. The antibody used in this study reacts with all members of the Src kinase family; Blk, Fgr, Fyn, Hck, Lck, Lyn, Src and Yes [18]. We have previously observed that FYN, one of the Src family kinases, is important for growth and survival of our MCF-7-derived tamoxifen resistant cell lines [41], and that the broad-spectrum Src family kinase inhibitor PP2 exerted preferential growth inhibition of the tamoxifen resistant MCF-7 breast cancer cell lines compared to the parental cells [41].

The modest effect of dasatinib on parental cell growth compared to the substantial growth inhibition seen with the resistant T47D breast cancer cell lines in the presence of their antiestrogens, suggests that growth of the resistant cells are more dependent on kinase-mediated signaling pathways than parental cells [13]. In this study, we were unable to immunoprecipitate Src with the HER receptor antibodies in the two tamoxifen resistant cell lines. This indicates lack of strong association between Src and the HER receptors and is in contrast to the fulvestrant resistant T47D cells, where we see an association between Src and the HER receptors [5]. Src may therefore be driving growth of these tamoxifen resistant breast cancer cell lines independent of the HER receptors e.g. via PDGFR or VEGFR. The observed detachment of the antiestrogen resistant cells after dasatinib treatment may be the result of Src-mediated signaling to the adhesion-associated protein kinase FAK (focal adhesion kinase) [18], which previously has been described to be activated in tamoxifen resistant breast cancer cells [42].

Although multiple mechanisms for resistance to antiestrogen therapy have been identified in preclinical studies, no effective treatment regime has been developed to overcome resistance in patients. Certainly, tumor heterogeneity and alternative survival pathways activated during drug treatment make the treatment of breast cancer patients with recurrent disease a complex and major clinical challenge and may require combination of treatment against different targets. Thus, therapies combining both endocrine and non-endocrine agents may have a better therapeutic effect than single agent treatment in tumors with functional ER. Tamoxifen resistant T47D cell lines maintain expression of ER and are growth inhibited by fulvestrant treatment, indicating that ER-signaling still is important for growth of these cell lines. The complete growth arrest observed after combined treatment with dasatinib and fulvestrant suggest that both ER and Src kinases are important and distinct contributors to growth of tamoxifen resistant T47D cells. In a recent study, we have shown that ER plays an important role for growth of ER-positive tamoxifen resistant MCF-7 cell lines [35]. Treatment of the tamoxifen resistant MCF-7 cells with the multi-targeting kinase inhibitors sorafenib and nilotinib restored the sensitivity to tamoxifen, whereas combined treatment with fulvestrant did not result in further growth inhibition [43]. This suggests that sorafenib and nilotinib target ER as well as other important growth promoting pathways, and indicates that growth of the tamoxifen resistant MCF-7 cells depends on kinase-activated ER activity. Based on our results and the above mentioned recent published data, we believe that treatment of tamoxifen resistant breast cancer with a functional ER should be a combination of fulvestrant and an ER-independent kinase inhibitor or by tamoxifen in combination with a kinase inhibitor targeting ER activation as well as other important growth promoting pathways. This is in line with a recent study showing that dasatinib and fulvestrant in combination exhibited synergistic effects in ER-positive MCF-7/LTED xenografts leading to complete block of tumor cell invasion and consequentely prevention of metastatic disease [13]. Notably, our tamoxifen resistant T47D cells were 10–20% growth-stimulated in the presence of tamoxifen, and propagation of the tamoxifen resistant cells in the absence of tamoxifen resulted in lower growth rate compared to resistant cells propagated in the presence of tamoxifen. This indicates an agonistic effect of tamoxifen on cell growth as demonstrated in our MCF-7-derived tamoxifen resistant breast cancer cells [35].

In contrast to the tamoxifen resistant T47D breast cancer cell lines, growth of the ER-negative fulvestrant resistant cells is different. As expected, treatment with tamoxifen alone had no effect on cell growth compared to untreated control, and dasatinib and tamoxifen in combination had no additional effect on cell growth compared to treatment with dasatinib alone. Thus, other growth stimulating mechanisms independent of both ER and Src may be driving part of the fulvestrant resistant cell growth.

In this study, immunohistochemical analysis performed on primary breast tumors from tamoxifen-treated patients revealed that Src at the plasma membrane was an independent biomarker for early recurrence and death. The antibodies used targeted all Src receptor family members and therefore no distinction between the Src members and survival could be made. Previous studies have described associations between Src located in the cytoplasm and decreased breast cancer survival [17,40,44], whereas nuclear Src is linked to a better prognosis [40,45]. It has been documented that Src located at the plasma membrane is active and necessary for promoting HER receptor complex formation which leads to increased cancer cell growth and survival [37]. In this study, we found that expression of Src at the plasma membrane was associated with poor disease-free and overall survival of tamoxifen treated breast cancer patients. We were, however; not able to identify any significant association between expression of activated Src and recurrence or death. It is well-known that phosphorylated proteins may be unstable [46] and our findings that only 13.4% of the tumors expressed phosphorylated Src whereas 57.5% expressed Src at the plasma membrane indicate that phosphorylated Src is unstable and difficult to detect and may explain the lack of correlation to clinical outcome. We identified an association between Src expressed at the plasma membrane and active HER receptors in the breast tumors. Thus, further investigations into the role of Src as a biomarker for relapse on tamoxifen alone or in combination with expression of the active forms of the HER receptors are warranted.

Endocrine resistance occurs via multiple mechanisms some of which are not fully clarified. However, understanding the key pathways for resistance and targeting these seem essential to improve survival of breast cancer patients. Our data show that ER and Src are important drivers of growth of tamoxifen resistant cells, whereas growth of fulvestrant resistant cells is ER independent and requires signaling via Src and yet unknown pathways. The finding that total growth arrest and cell death of tamoxifen resistant cell lines were obtained with fulvestrant in combination with a Src inhibitor support that tamoxifen treatment should preceed fulvestrant therapy, which leads to ER independent growth. Our clinical analyses revealed that Src is a biomarker for reduced benefit from tamoxifen treatment, and suggest that Src may be a potential biomarker to select tamoxifen resistant patients with ER-positive tumors, who will benefit from fulvestrant therapy in combination with a Src inhibitor.
